# Lithium Intercalation into the Excitonic Insulator Candidate Ta_2_NiSe_5_

**DOI:** 10.1021/acs.inorgchem.3c01510

**Published:** 2023-07-19

**Authors:** P. A. Hyde, J. Cen, S. J. Cassidy, N. H. Rees, P. Holdship, R. I. Smith, B. Zhu, D. O. Scanlon, S. J. Clarke

**Affiliations:** † Department of Chemistry, Inorganic Chemistry Laboratory, University of Oxford, South Parks Road, Oxford OX1 3QR, U.K.; ‡ Department of Chemistry, University College London, 20 Gordon Street, London WC1H 0AJ, U.K.; § Thomas Young Centre, University College London, Gower Street, London WC1E 6BT, U.K.; ∥ Department of Earth Sciences, University of Oxford, South Parks Road, Oxford OX1 3AN, U.K.; ⊥ Rutherford Appleton Laboratory, ISIS Facility, Harwell Campus, Didcot, Oxon OX11 0QX, U.K.

## Abstract

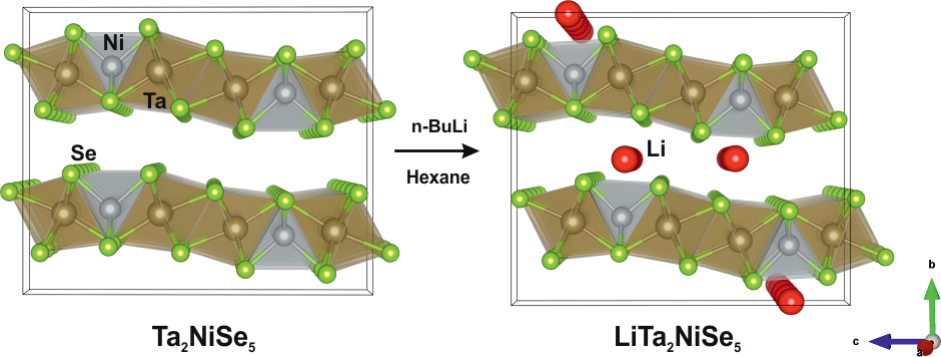

A new reduced phase derived from the excitonic insulator candidate Ta_2_NiSe_5_ has been synthesized via the intercalation of lithium. LiTa_2_NiSe_5_ crystallizes in the orthorhombic space group *Pmnb* (no. 62) with lattice parameters *a* = 3.50247(3) Å, *b* = 13.4053(4) Å, *c* = 15.7396(2) Å, and *Z =* 4, with an increase of the unit cell volume by 5.44(1)% compared with Ta_2_NiSe_5_. Significant rearrangement of the Ta-Ni-Se layers is observed, in particular a very significant relative displacement of the layers compared to the parent phase, similar to that which occurs under hydrostatic pressure. Neutron powder diffraction experiments and computational analysis confirm that Li occupies a distorted triangular prismatic site formed by Se atoms of adjacent Ta_2_NiSe_5_ layers with an average Li–Se bond length of 2.724(2) Å. Li-NMR experiments show a single Li environment at ambient temperature. Intercalation suppresses the distortion to monoclinic symmetry that occurs in Ta_2_NiSe_5_ at 328 K and that is believed to be driven by the formation of an excitonic insulating state. Magnetometry data show that the reduced phase has a smaller net diamagnetic susceptibility than Ta_2_NiSe_5_ due to the enhancement of the temperature-independent Pauli paramagnetism caused by the increased density of states at the Fermi level evident also from the calculations, consistent with the injection of electrons during intercalation and formation of a metallic phase.

## Abstract 2

LiTa_2_NiSe_5_ is a new reduced metallic phase synthesized from the layered ternary chalcogenide and excitonic insulator candidate Ta_2_NiSe_5_. Intercalation of lithium causes unit cell expansion, significant rearrangement of the Ta_2_NiSe_5_ layers, and suppression of the monoclinic distortion observed in Ta_2_NiSe_5_. Li occupies a single trigonal prism site, and the net diamagnetism is reduced relative to Ta_2_NiSe_5_.

## Introduction

Over 60 binary metal chalcogenides are known, and over two-thirds form layered compounds as a consequence of the high polarizability of the chalcogenide ions.^1^ A surge in the synthesis and characterization of ternary chalcogenides in the 1980s^2−9^ resulted from the development of air-sensitive synthesis techniques and improved characterization methods. Since then, transition metal chalcogenides have been studied extensively due to their unique physical properties and diverse structural chemistry. Of these, systems exhibiting low dimensionality are considered prime candidates for intercalation. Intercalation of Li into TiS_2_ to form Li_*x*_TiS_2_ was used as a prototypical secondary battery system,^10^ and insertion of atoms into the van der Waals gap of layered materials can be used to alter their physical properties, for example, the enhancement of the superconducting *T*_c_ from 8 to 45 K through intercalation of Li and NH_3_ into the superconducting layered chalcogenide FeSe^11^ or the realization of superconductivity in the excitonic insulator candidate 1*T*-TiSe_2_ through the intercalation of Cu, with a superconducting *T*_c_ of 4.15 K.^12^

**Figure 1:**
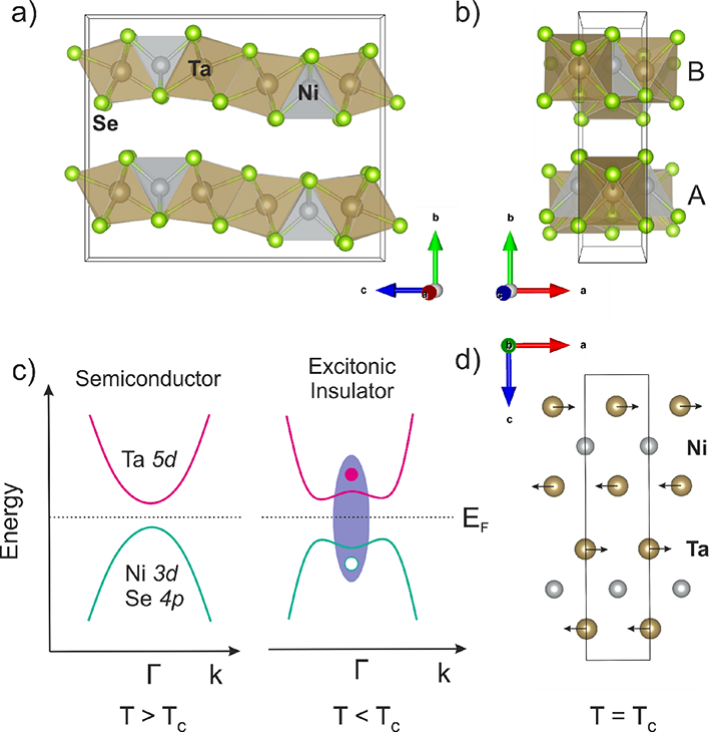
(a) Structure of Ta_2_NiSe_5_ viewed along the *a*-axis and (b) Ta_2_NiSe_5_ viewed along the *c*-axis demonstrating the AB-layer stacking of the layers, which are offset from one another along the *a*-axis. (c) Schematic of the band dispersion of Ta_2_NiSe_5_ in the semiconducting state and as an excitonic insulator. The purple shaded area shows the bound hole–electron pair. Figure adapted from Kim *et al.*(18) (d) Schematic representation showing the lattice distortion of the 1D Ta chains relative to the Ni chains corresponding to the orthorhombic to monoclinic phase transition.

The narrow-band-gap semiconductor Ta_2_NiSe_5_ is a candidate for being an excitonic insulator.^13,14^ In such a proposed state arising in narrow-band-gap semiconductors such as Ta_2_NiSe_5_ or 1*T*-TiSe_2,_^15,16^ excitons are formed spontaneously if the exciton binding energy is less than the band gap, and because the electron and hole pairs are strongly bound, the state is insulating and may be thought of as an unconventional insulating state. Since the formation of the excitonic insulating phase arises from the formation of strongly bound charge-neutral pairs, the transition can be understood as a Bose–Einstein condensation (BEC). A schematic of the band dispersion of the narrow-gap semiconducting and excitonic insulating states is shown in Figure 1c.^17^ Structurally, Ta_2_NiSe_5_ is a layered compound constructed from Ta_2_NiSe_5_ layers stacked along the *b*-axis and related by the *C*-centering translation so that an AB-type stacking results as shown in Figure 1a,b.^8^

The layers comprise octahedral TaSe_6_ and tetrahedral NiSe_4_ polyhedra joined via edge sharing. The layers are gently corrugated along the *c* direction, and along the *a* direction, there are ribbons of double-thickness edge-shared TaSe_6_ octahedra linked by chains of vertex-linked NiSe_4_ tetrahedra. Resistivity measurements reveal semiconductor behavior at room temperature and a gradual transition to a state where the resistivity behaves like that of a metal above 550 K.^19^ An additional feature at 328 K is accompanied by a structural phase transition from *Cmcm* to *C*2/*c* symmetry on cooling. Structurally, this is equivalent to the shearing of the Ta chains against the Ni chains, as shown schematically in Figure 1d. Chemically, the composition is intriguing, with the possibility of Ta(IV)/Ni(II) versus Ta(V)/Ni(0) configurations seemingly plausible. Chemical intuition would argue for the former, but the observed diamagnetism is not consistent with Ni(II) and the fact that there is no structural evidence for a charge-density wave (CDW) distortion associated with the Ta(IV) 5d^1^ configuration that would account for the observed insulating behavior (rather than metallicity) seemingly argues for the latter. Tight-binding band structure calculations of the *Cmcm* phase indicate that Ta_2_NiSe_5_ has a direct gap at the Γ point of the Brillouin zone.^20,21^ The conduction band is largely configured by the Ta 5d_*xy*_ chain giving a quasi-1D dispersion, while the top of the valence band also has a quasi-1D dispersion and is composed of well-hybridized Ni 3d_*xz*_ and Se 4p_*y*_ orbitals. There is no mixing of the states at the top of the Ni/Se-based valence band and the bottom of the Ta-based conduction band because the states belong to different irreducible representations at Γ, and the appropriate formulation is Ta(V) and Ni(0). Canadell and Whangbo^20^ point out that the Ta–Ta separations are long (consistent with Ta(V)), while the Ta–Ni separation is short, suggesting that the Ni(0) oxidation state is stabilized by donation to the empty Ta(V) acceptor orbitals analogous to the stabilization of Ni(0) in molecules such as Ni(CO)_4_. Kaneko *et al*.^22^ account for the phase transition from the *Cmcm* phase to the *C*2/*c* phase as driven by the BEC of excitonic electron–hole pairs, while a more recent report suggests that spontaneous symmetry breaking is mostly structural in nature.^23^ This symmetry-lowering distortion accompanying the formation of the excitonic insulator phase allows mixing of the conduction and valence bands at Γ, and this mixing is proposed^24^ to account for the observation of flattening of the bands around Γ in ARPES experiments and the interpretation of the ARPES data using an electronic description, which suggests a Ta(IV) d^1^ state and an oxidized Ni 3d^9^/Se-ligand-hole state.^22,24,25^ Further experiments devoted to fully understanding the transition are the topic of current research.^26^

High-pressure experiments^27^ on Ta_2_NiSe_5_ reveal the sensitivity of the structure to applied pressure. The ambient temperature and pressure phase crystallizing in *C*2/*c* and assigned as the excitonic insulator phase transforms to the *Cmcm* phase under an applied hydrostatic pressure of about 2 GPa at ambient temperature. This *Cmcm* phase is the high-temperature/ambient-pressure high-symmetry phase described above, and it transforms at about 3 GPa at ambient temperature to a *Pmnm* phase related to the *Cmcm* phase by a relative sliding of the layers to produce an AA stacking (rather than AB stacking) of the layers with the loss of lattice centering. This high-pressure orthorhombic phase also undergoes a transition on cooling at high pressure to a monoclinic phase crystallizing in *P*2/*n*, and Nakano *et al.*(27) suggest that this may also be an excitonic insulator phase. Superconductivity below 1.2 K was recently found at 8 GPa in this high-pressure phase of Ta_2_NiSe_5,_^28^ which is reminiscent of the pressure-induced superconductivity found in the excitonic insulator candidate 1T-TiSe_2_.^29^

Chemical transformation by intercalation of lithium into Ta_2_NiSe_5_ has previously been reported by Squattrito *et al.*(30) using *n*-butyllithium to produce a compound of formula Li_2_Ta_2_NiSe_5_. However, the powder X-ray diffraction (PXRD) pattern was not successfully indexed, apparently due to the low crystallinity of the product. Here, we present the reduced phase LiTa_2_NiSe_5_ synthesized using chemical intercalation and characterized using high-resolution PXRD, powder neutron diffraction (PND), nuclear magnetic resonance spectroscopy (NMR), and computational studies. The intercalation results in significant structural and electronic changes compared with the host, and here, we compare and contrast this reduced phase with the ambient^8^ and high-pressure forms of the host Ta_2_NiSe_5_.^27^

## Experimental Section

### Synthesis

All syntheses were carried out in a Glove Box Technology Ltd. argon-filled dry glovebox with an O_2_ content below 1 ppm or on a Schlenk line under N_2_. Polycrystalline samples of Ta_2_NiSe_5_ were synthesized by grinding together tantalum powder (Alfa Aesar, 99.97%), nickel powder (Alfa Aesar, 99.9%), and selenium powder (Alfa Aesar, 99.999%) in stoichiometric amounts using an agate pestle and mortar until the mixture was homogeneous. The mixture was then sealed inside an evacuated silica tube and heated at 750 °C for 7 days (ramping rate of 5 °C min^–1^) before being allowed to cool naturally. The resulting powder was reground and pressed into a pellet before reheating to 700 °C for 48 h and cooling at the natural rate of the furnace. This second step was found to improve the crystallinity of the precursor Ta_2_NiSe_5_.

Lithium intercalates were synthesized by inserting Ta_2_NiSe_5_ powder and a magnetic stirrer bar into a Schlenk tube in the glove box. Approximately 20 cm^3^ of dry hexane was then added to the vessel using a Schlenk line. A stoichiometric volume of *n*-butyllithium solution (1.6 M in hexanes; Alfa Aesar) to give the target phase LiTa_2_NiSe_5_ was added using a nitrogen-purged needle and syringe. The suspension was left to stir overnight before filtering off the supernatant using a Schlenk line. The solid was then washed by adding fresh dry hexane to the Schlenk tube, stirring for 30 s, and removing the solvent by filtration. This procedure was repeated twice. The remaining solid was left to dry under dynamic vacuum for 1 h.

### Diffraction

Laboratory PXRD data on the solid products were collected using a Bruker D8 Advance Eco diffractometer (Cu Kα radiation). For detailed structural refinement, data were collected on beamline I11^31^ at the Diamond Light Source using 1.5 min scans with a MYTHEN position sensitive detector (PSD), using Si-calibrated 0.82 Å X-rays. The PSD was also used to gather diffraction patterns at 82 temperatures over a range of 100–300 K and 211 temperatures over a range of 303–1173 K. PND measurements at ambient temperature were made using the GEM^32^ instrument at the ISIS Pulsed Neutron and Muon Facility, Rutherford Appleton Laboratory, UK. A sample of approximately 1.5 g was loaded into a 6 mm-diameter vanadium can and sealed using an indium gasket. Diffraction data were collected for an integrated proton charge to the ISIS target of 350 μAhr (microamp hours), equivalent to approximately 2 h exposure in the neutron beam. Refinements of the structural models against the diffraction data were carried out using TOPAS Academic V6 software.^33^

### Magnetometry

Magnetic susceptibility measurements were made using a Quantum Design MPMS-3 SQUID magnetometer. Accurately weighed samples of around 30 mg were loaded into gelatin capsules, which were secured inside plastic straws, which were loaded into the instrument. Zero-field-cooled (ZFC) and field-cooled (FC) measurements were carried out in fields of 2.5 and 3.5 T. High fields were necessary in order to measure these materials, which have a very low magnetic susceptibility. An additional ZFC plus FC measurement was performed in a field of 50 Oe to check for a possible superconducting transition.

### NMR Spectroscopy

Solid-state ^6^Li NMR measurements were made using a Bruker Avance III-HD spectrometer equipped with a 9.4 T wide-bore magnet and a 4 mm magic angle spinning (MAS) probe. Samples were packed in 4 mm-outer-diameter (OD) rotors for measurements below 298 K and in sealed inserts, which were then placed in a 4 mm-OD rotor for measurements above 298 K. This was to alleviate tuning and spinning problems caused by a phase transition apparent just above 298 K. For low-temperature measurements, a MAS rate of 8 kHz was used, and spectra were acquired using a one-pulse excitation sequence, 500 scans, and a relaxation delay of 18 s. For the high-temperature experiments, a MAS rate of 10 kHz was used and the same conditions as the low-temperature experiments except that 3500 scans were acquired. Spectra were externally referenced to LiCl in H_2_O (0 ppm).

### Chemical Analysis

Inductively coupled plasma-mass spectroscopy (ICP-MS) was used to determine the Li content of the washed samples. Approximately 5 mg of the sample was dissolved in 20 mL of HNO_3_ (conc.):HCl (12 M) in a 19:1 volume ratio and diluted with deionized water to make a 2 vol % solution of the original concentrated acids. A PerkinElmer NexION 350D ICP-MS at the Department of Earth Sciences, University of Oxford, was used to determine the concentrations of Li and Ni.

### Computation

The plane wave density functional theory (DFT) code Vienna ab initio simulation package (VASP)^34−36^ (version 5.4.4) was used to determine lithium site energies. A plane wave cutoff energy of 600 eV and Γ-centered Monkhorst–Pack (MP) grids with a maximum spacing of 0.043 2π Å^–1^ were used with projector-augmented wave^37,38^ (PAW) pseudopotentials from dataset PBE.54 (Li_sv, Ta_pv, Ni_pv, and Se). The PBEsol^39^ generalized gradient approximation (GGA) and dispersion-corrected^40^ PBEsol + D3 functionals were used to model the exchange and correlation effects. Tolerances of 10^–6^ eV and 10^–2^ eV Å^–1^ were applied to total energy and forces during electronic minimization and geometry optimization, respectively. The electronic densities of states (DOS) were computed using denser *k*-point grids with a maximum spacing of 0.025 2π Å^–1^. Analysis of the DOS was conducted using the sumo^41^ Python package.

Input files for lithium site calculations were prepared using the doped^42,43^ Python package with a 32-atom supercell. Force tolerance was raised to 2 × 10^–2^ eV Å^–1^ for lithium interstitials during relaxations, with fixed lattice parameters. The pymatgen^44^ and ase^45^ Python packages were used to manipulate structures and to assist with the symmetry analysis of the lithium sites.

## Results and Discussion

### Structural Refinement

**Figure 2:**
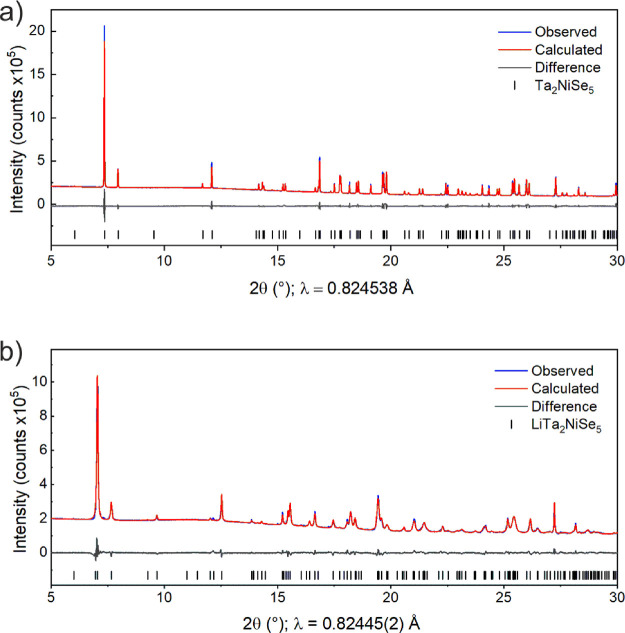
Fitted PXRD patterns of (a) Ta_2_NiSe_5_ and (b) LiTa_2_NiSe_5_ measured at 300 K on the PSD detector at I11 showing the observed (blue), calculated (red), and difference (gray) curves. *R*_wp_: 2.23 and 1.76%, respectively.

**Figure 3:**
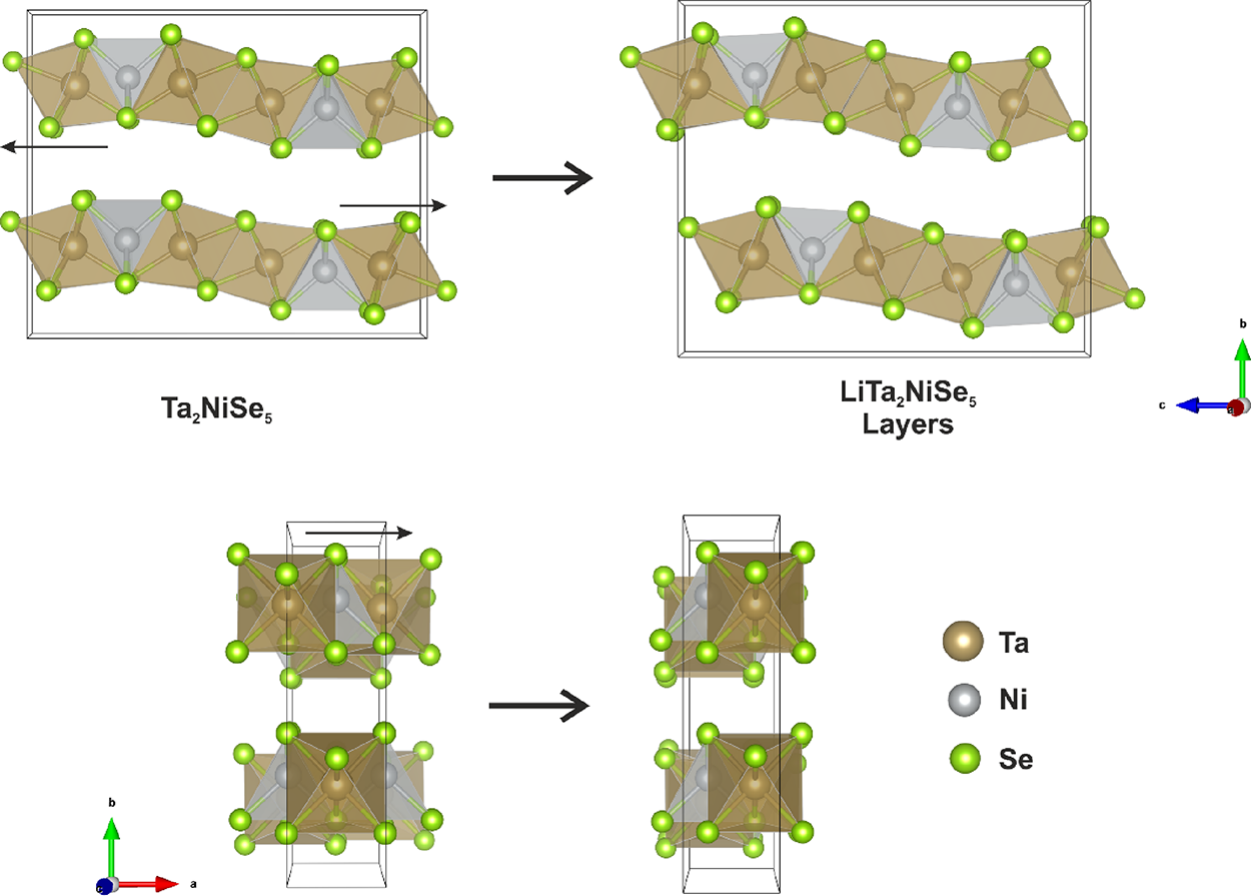
(Left) Crystal structure of Ta_2_NiSe_5_. (Right) Crystal structure of the Ta–Ni–Se slabs of the Li intercalate in *Pmnb*. Small black arrows represent the displacement of the alternate layers by (0.5, 0, −0.1372(3)) upon intercalation.

The PXRD pattern of the intercalate had visual similarities to that of the parent compound Ta_2_NiSe_5_, shown in Figure 2. The pattern could be well-indexed to an orthorhombic unit cell, *a* = 3.502 Å, *b* = 13.405 Å, and *c* = 15.739 Å, with a fairly similar shape to that of Ta_2_NiSe_5_ but with an increase in the unit cell volume of 5.44(1)%. Observation of the systematic absences in the pattern indicated the extinction symbol P*nb with this set of axes. A rigid-body approach to the structure solution was adopted to take advantage of the layered nature of the structure with the assumption that the layers would remain intact during the low-temperature intercalation experiments. A model resembling the parent phase Ta_2_NiSe_5_ in terms of the arrangement of the atoms and the interatomic distances was built in *P*1 symmetry inside a unit cell with the unit cell parameters obtained from the indexing described above, with the *b*-axis being the stacking direction. The atoms were then divided into two groups according to which layer they occupied and constrained, so there was no refinement of relative atomic positions within each layer. One layer was kept fixed, and the position of the second layer relative to the first was refined in the *x*, *y*, and *z* directions. The resulting displacement of the two layers relative to their positions in the parent structure refined to (0.5(1), −0.0156(4), −0.1372(3)). The displacement in *x* (i.e., along the short axis) is within an error of 0.5 and was assumed to be so for the purpose of this model, so there is a substantial relative shift in this direction. The small displacement in *y* was fixed to zero. Although this was outside the estimated uncertainty, a value of zero means that the two layers remain displaced by 1/2*b* along the *b*-axis, which is the stacking direction, consistent with the expectation that they remain evenly spaced. Compared with the situation in Ta_2_NiSe_5_, the layers are displaced slightly relative to one another along the *c* direction. The final relative arrangements of the layers in the host and intercalate compounds are compared in Figure 3. The resulting structure of the intercalate obtained from this rigid-body refinement in *P*1 can then be described in *Pmnb* (a setting of space group no. 62), consistent with the analysis of systematic absences. The final model was thus produced by completely lifting the rigid-body constraints and allowing atomic positions to freely refine in the *Pmnb* cell. An alternative model in the space group *P*2_1_/*n* (a setting of space group no. 14 with the *b*-axis unique, allowing a direct symmetry descent from *Pmnb*) was generated to assess whether a small monoclinic distortion akin to that in Ta_2_NiSe_5_ was present. No clear monoclinic distortion was observed (β = 90.01(1)°), and no improvement to the fit could be achieved in *P*2_1_/*n* (*R*_wp_ = 2.26%) versus *Pmnb* (*R*_wp_ = 1.76%). The *Pmnb* model was accepted on this basis. The final structural model is shown in Figure 2 with refinement parameters given in Table 1.

**Table 1:** Refinement Parameters from the PXRD and PND Patterns of LiTa_2_NiSe_5_^a^

LiTa_2_NiSe_5_: RMM = 822.33 g mol^–1^, *Z* = 4	
diffractometer	I11 (PSD) (GEM(ISIS))
wavelength (Å)	0.82445(2) (ToF)
temperature (K)	300 (300)
space group	*Pmnb* (62)
*a* (Å)	3.50247(3) (3.49523(6))
*b* (Å)	13.4053(4) (13.3681(3))
*c* (Å)	15.7396(2) (15.7015(4))
*V* (Å^3^)	739.002(27) (733.644(28))

atom	site	*x*	*y*	*z*	occ	*U*_iso_ (Å^2^)
Ta1	4*c*	0.25 (0.25)	0.2304(2) (0.2256(3))	0.0389(2) (0.0404(3))	1	0.0206(9) (0.0133(11))
Ta2	4*c*	0.25 (0.25)	0.2072(2) (0.2089(3))	0.3213(2) (0.3204(3))	1	0.0148(7) (0.0075(9))
Ni	4*c*	0.25 (0.25)	0.2999(4) (0.3051(2))	0.6850(5) (0.6803(2))	1	0.0050(16) (0.0109(7))
Se1	4*c*	0.25 (0.25)	0.9081(4) (0.9073(3))	0.9411(3) (0.9388(2))	1	0.0270(19) (0.0067(8))
Se2	4*c*	0.25 (0.25)	0.9245(4) (0.9279(3))	0.7099(3) (0.7082(2))	1	0.0237(21) (0.0118(10))
Se3	4*c*	0.25 (0.25)	0.1592(4) (0.1639(3))	0.8752(3) (0.8776(2))	1	0.0073(18) (0.0060(8))
Se4	4*c*	0.25 (0.25)	0.1502(4) (0.1415(3))	0.4800(3) (0.4814(2))	1	0.0150(19) (0.0122(10))
Se5	4*c*	0.25 (0.25)	0.3136(3) (0.3139(3))	0.1847(4) (0.1858(2))	1	0.0048(13) (0.0098(8))
Li	4*c*	(0.25)	(0.506(9))	(0.308(7))	(1.04(7))	(0.180(19))

a Refinement parameters from the PND pattern of LiTa_2_NiSe_5_ collected at 300 K on the GEM instrument at ISIS by simultaneous fitting to data collected in all 6 detector banks are given in parentheses.

The PXRD data show broadening of the diffraction peaks indicating a noticeable reduction in the crystallinity between Ta_2_NiSe_5_ and LiTa_2_NiSe_5_, which we attribute to reduction in particle size during the intercalation step. Grain sizes calculated using the Scherrer equation are 150(3) and 100(5) nm for Ta_2_NiSe_5_ and LiTa_2_NiSe_5_, respectively. The ratios of the full-width at half-maximum (FWHM) of well-resolved *h*0*l* and 0*k*0 reflections were found to be 1.13 and 0.95 for Ta_2_NiSe_5_ and LiTa_2_NiSe_5_, respectively, indicating that the peak broadening is fairly isotropic. This further supports our hypothesis that peak broadening is an effect of the reduced particle size.

An additional sample was synthesized using a 2:1 ratio of *n*-butyllithium to Ta_2_NiSe_5_ to replicate the method used by Squattrito *et al*.^30^ This sample had poor crystallinity as reported, and the PXRD pattern only displayed two well-resolved reflections. We propose that the use of additional *n*-BuLi may generate a Li-richer phase but with reduced crystallinity.

### Powder Neutron Diffraction (PND)

**Figure 4:**
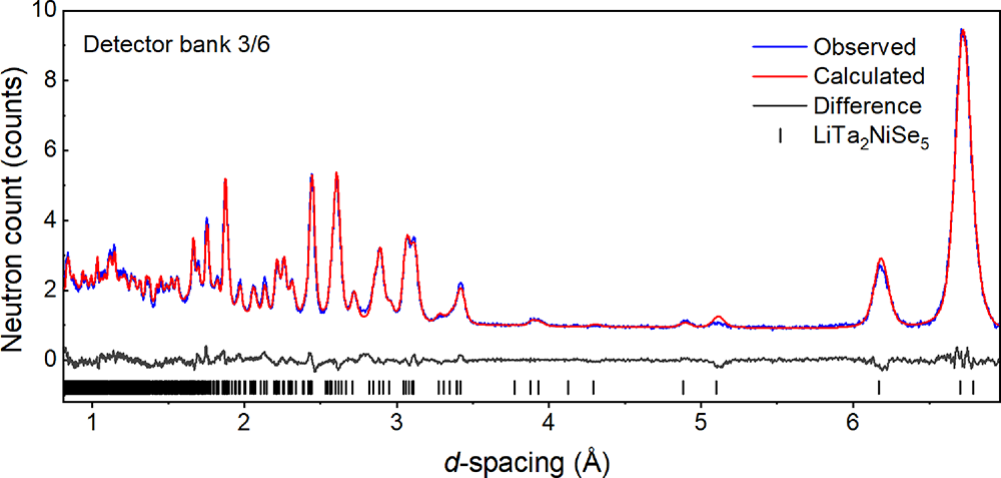
Fitted PND pattern of LiTa_2_NiSe_5_ measured at 300 K on the GEM instrument at ISIS (bank 3 with the range 24 < 2θ < 45°) showing the observed (blue), calculated (red), and difference (gray) curves. *R*_wp_: 4.58%. Rietveld fits to the patterns collected in all 6 detector banks are presented in Figure S1 in the Supporting Information.

PND experiments were performed in order to attempt to locate the light Li atoms. The Rietveld refinement against room temperature data from GEM (Figure 4) was consistent with the *Pmnb* model generated from the PXRD data given in Table 1. The only scattering center with a negative scattering amplitude given by the difference Fourier map, consistent with Li, in the interlayer spacing is located at (0.25, 0.506(9), 0.308(7)), a distorted triangular prismatic site formed by Se atoms of adjacent Ta_2_NiSe_5_ layers. The Li occupancy was refined to be 1.04(7) and is consistent with the stoichiometry of the reaction, the high phase purity of the intercalate product, and the results of ICP-MS measurements of the washed solid sample, which gave a Li:Ni ratio of 0.96(4). The stoichiometry has been assigned as LiTa_2_NiSe_5_ from these independent measurements, which are consistent with the reaction stoichiometry. The thermal displacement parameter of Li from the refinement is 0.180(19) Å^2^, larger than for any other atom in the model by one order of magnitude and which could be a result of high mobility of Li or due to a combination of the low scattering length of Li (−1.90 fm), compared with those of the other atoms (Ta: 6.91 fm, Ni: 10.3 fm, and Se: 7.97 fm) and the large number of parameters required to fully describe the structure.

**Figure 5:**
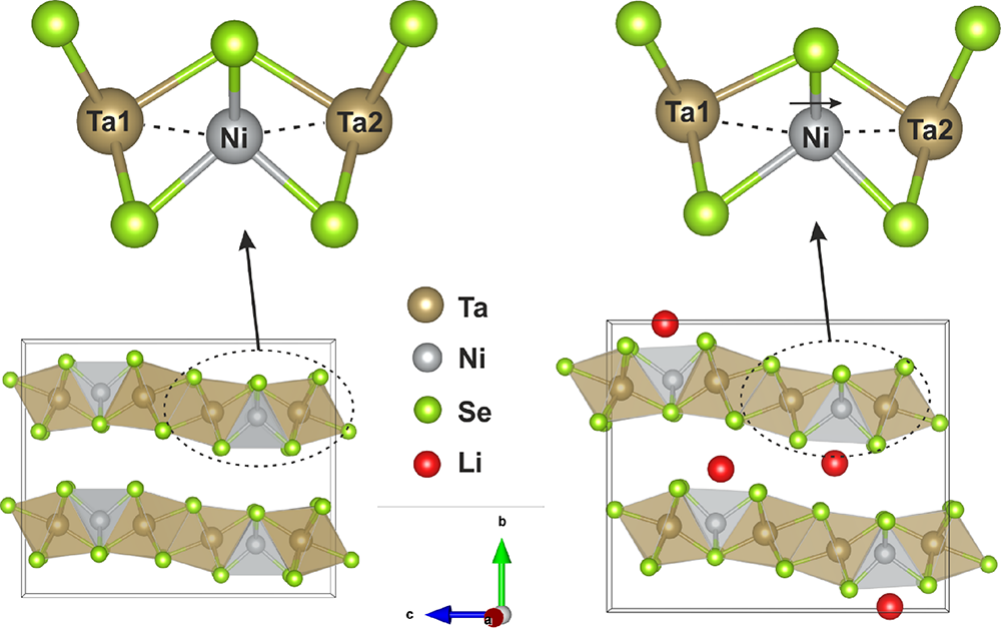
Coordination environment surrounding Ni in (left) Ta_2_NiSe_5_ and (right) LiTa_2_NiSe_5_. Ta1-Ni and Ta2-Ni distances are represented with the dashed line.

The calculated metal–metal separations for Ta_2_NiSe_5_ and LiTa_2_NiSe_5_ are listed in Table 2. The values obtained for Ta_2_NiSe_5_ from PXRD data are within one standard deviation of those reported by Sunshine and Ibers from single-crystal XRD data.^8^ Canadell and Whangbo^20^ comment on the long Ta–Ta and short Ta–Ni separations in Ta_2_NiSe_5_ supporting the Ta(V)/Ni(0) oxidation state configuration. Both Ta1–Ni and Ta2–Ni distances in Ta_2_NiSe_5_ are approximately equal at 2.8037(8) and 2.8131(8) Å, respectively. In LiTa_2_NiSe_5_, the structure refinements and the computation both suggest that the Ta1–Ni separation is somewhat elongated relative to the Ta2–Ni separation (Figure 5), although the difference is only significant in the PXRD data (2.947(7) and 2.740(7) Å, respectively) for which Ni makes a relatively weak contribution to the scattering compared with the PND data, so this may be an artifact. The partial density of states calculated for each Ta site, discussed below, suggests that both sites are reduced equally and the subtle differences in band structure are due to the different local coordination around the two Ta sites.

**Table 2:** Calculated Bond Distances of Ta_2_NiSe_5_ and LiTa_2_NiSe_5_ from PXRD, PND, and Computed Models Determined in This Work^a^

compound	Ta_2_NiSe_5_	LiTa_2_NiSe_5_		
distances (Å)	PXRD	PXRD	PND	computation
Ta1–Ta1	3.496(1)	3.5025(1)	3.4952(1)	3.4781
Ta1–Ta2	3.903(1)	3.943(4)	3.969(6)	3.9268
Ta1–Ni	2.8037(8)	2.947(7)	2.837(5)	2.8479
Ta2–Ni	2.8131(8)	2.740(7)	2.816(5)	2.7890
Ta1–Se1	2.523(1) [Se1]	2.587(6)	2.512(5)	2.5650
Ta1–Se3	2.678(1) [Se2]	2.761(6)	2.686(6)	2.6798
Ta1–Se4	2.588(1) [Se2]	2.546(5)	2.659(5)	2.6173
Ta1–Se5	2.570(1) [Se3]	2.572(7)	2.570(6)	2.5662
Ta2–Se2	2.581(1) [Se1]	2.529(5)	2.569(5)	2.5602
Ta2–Se3	2.661(1) [Se2]	2.660(5)	2.598(5)	2.6078
Ta2–Se4	2.678(1) [Se2]	2.628(6)	2.684(6)	2.6787
Ta2–Se5	2.570(1) [Se3]	2.553(7)	2.537(6)	2.5428
Ni–Se1	2.339(2) [Se1]	2.456(9)	2.365(3)	2.3329
Ni–Se2	2.339(2) [Se1]	2.348(9)	2.400(5)	2.3590
Ni–Se5	2.381(2) [Se3]	2.335(5)	2.365(3)	2.3893

a Atomic labels of Se atoms for Ta_2_NiSe_5_ data in the space group *C*2/*c* are given in square brackets, for comparison to LiTa_2_NiSe_5_ in the space group *Pmnb*.

### Variable-Temperature Diffraction

Temperature-resolved PXRD measurements between 100 and 300 K were performed on LiTa_2_NiSe_5_ to probe for any structural distortions analogous to the monoclinic distortion observed in Ta_2_NiSe_5_ at 328 K. These high-resolution measurements revealed no peak broadening or splitting down to 100 K, indicating that there was no comparable distortion in LiTa_2_NiSe_5_ down to this temperature. The lattice parameters and unit cell volume output from the sequential Rietveld fits to the PXRD patterns indicated no structural transitions or anomalies, shown in Figure S2. An additional measurement between 303 and 1173 K was performed to assess the decomposition of LiTa_2_NiSe_5_. It was found that no significant peak broadening occurs below the decomposition temperature. The film plot in Figure S3 shows that decomposition occurs at approximately 700–800 K with the disappearance of the high-intensity reflection at 2θ of ∼7.1°. Beyond this temperature, two new low-angle reflections emerge at 2θ of ∼6 and ∼6.7°. The PXRD pattern collected at 1173 K, shown in Figure S4, cannot be fitted using LiTa_2_NiSe_5_, Ta_2_NiSe_5_, or any other known ternary or binary phases made up of the constituent elements of the intercalated phase, and we hypothesize that the pattern is dominated by a new phase or a mixture of phases. The change is not reversed on cooling back to 300 K. The phases formed in this high-temperature change are subject to further investigation.

To assess the air stability of LiTa_2_NiSe_5_, a portion of the sample was left to oxidize in air overnight. The resulting phase was found to be almost exclusively Ta_2_NiSe_5_, with slightly diminished crystallinity compared to fresh Ta_2_NiSe_5_ with an estimated crystallite size of 70(5) nm, from analysis of synchrotron PXRD data (Figure S5). Additional side phases could not be identified; however, there is most likely a lithium-containing amorphous phase resulting from the aerial deintercalation of Li. The significant change in the layer stacking on intercalation and deintercalation indicates high mobility of the Ta–Ni–Se layers, and the changes are reversible without significant incorporation of stacking disorder.

### Computation

**Figure 6:**
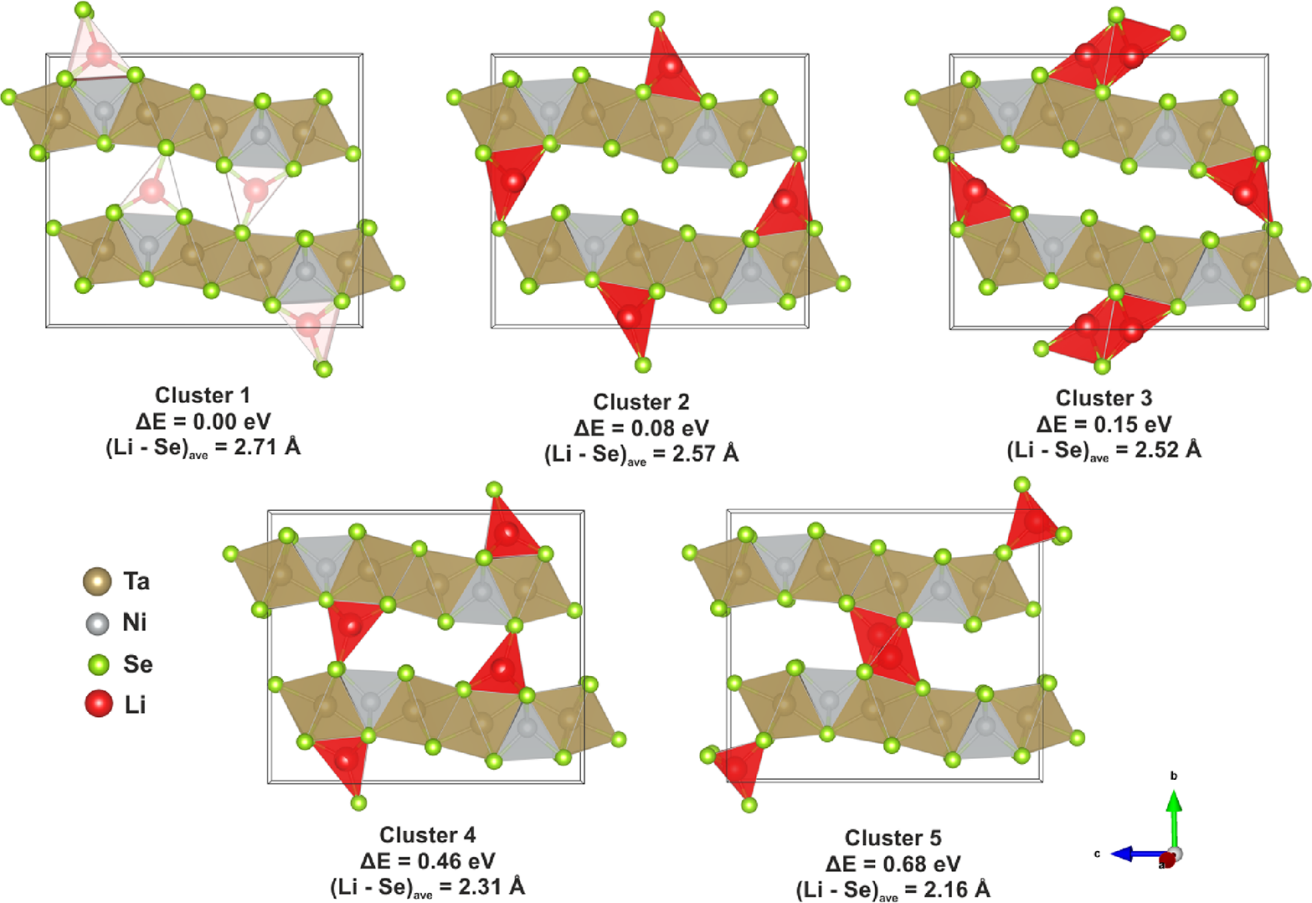
Schematic showing the five distinct clusters of Li sites determined by the plane wave density functional theory analysis and their relative energies. Positions for the lowest-energy site in each cluster can be found in Table 3 (column 2). All the sites determined by computation can be found in Table S2.

DFT calculations were performed on the *Pmnb* model to locate other possible Li sites and calculate their relative energies. Twenty-two symmetry-inequivalent interstitial positions were identified, and each Li interstitial was subject to relaxation in the host Ta_2_NiSe_5_ supercell with *Pmnb* symmetry using the PBEsol functional. All Li interstitials were relaxed to one of 5 different clusters of sites. The sites within each cluster have marginally different positions relative to one another but were found to be within 0.015 Å of each other along any given axis, a distance comparable to the uncertainty in a refined interatomic distance. Twenty-one of the individual sites were located on 4*c* sites of *m* site symmetry in the experimental *Pmnb* model. One additional site corresponded to a general position in *Pmnb* but was close to a mirror plane. Locations of all sites can be found in the Supporting Information (Table S2). The lowest-energy site belonging to each of the five clusters is shown in Figure 6, and their coordinates are given in Table 3. The Li site identified independently from the PND measurements with coordinates (0.25, 0.515(2), 0.332(3)) corresponds to the lowest-energy Li cluster, labeled as cluster 1 shown in Table 3. On this basis, the Li site identified through PND was accepted as the final model with an assigned occupancy of 1 consistent with the structure refinement and the chemical analysis.

**Table 3:** Parameters of the Lowest-Energy Interstitial Site of Each Cluster Output by Plane Wave Density Functional Theory Calculations^a^

				Li site coordinates		
defect name	Δ*E* (eV)	site	Li site energy (eV per Li)	*x*	*y*	*z*
cluster 1	0	4*c*	0	0.25	0.50114	0.33392
cluster 2	0.08	4*c*	0.02	0.25	0.46758	0.07313
cluster 3	0.15	4*c*	0.04	0.25	0.50076	0.92858
cluster 4	0.46	4*c*	0.11	0.25	0.57913	0.74604
cluster 5	0.68	4*c*	0.17	0.25	0.54079	0.51680

a Δ*E* is the total energy difference of the unit cell relative to the lowest-energy cluster model. The Li site energy is Δ*E* normalized by the number of Li atoms in the unit cell.

**Figure 7:**
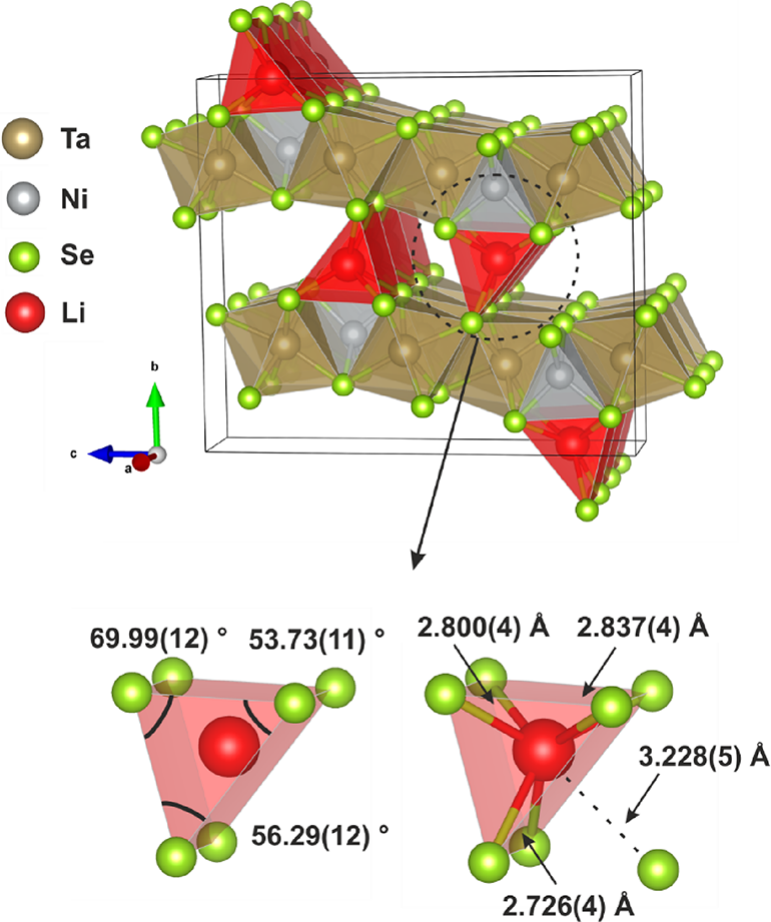
Final refined structural model of LiTa_2_NiSe_5_ from Rietveld fits to PXRD and PND and confirmed by computational prediction of the Li position. Bond lengths and angles are derived from fit to PND data.

The final structural model for LiTa_2_NiSe_5_ is shown in Figure 7. Li atoms lie in 6-coordinate distorted triangular prisms of Se with an average Li–Se bond length of 2.724(2) Å determined from the PND measurement. This is consistent with Li–Se bond length values reported in the literature, which range from 2.61 Å for the tetrahedral environment in Li_2_Se^46^ to 2.93 Å for 6-coordinate Li sites in LiMnSe_2_.^47^ An additional Se atom lies at a distance of 3.322(4) Å, which has not been formally included in the coordination sphere of Li since it lies far outside the range of typical values. The coordination environment of Li is also detailed in Figure 7, which gives the Li–Se bond distances and the angles of the LiSe_6_ polyhedra, indicating the degree of distortion away from a regular triangular prism. We propose that the stacking rearrangement of the Ta–Ni–Se layers is driven by the coordination requirements of the Li ions.

**Figure 8:**
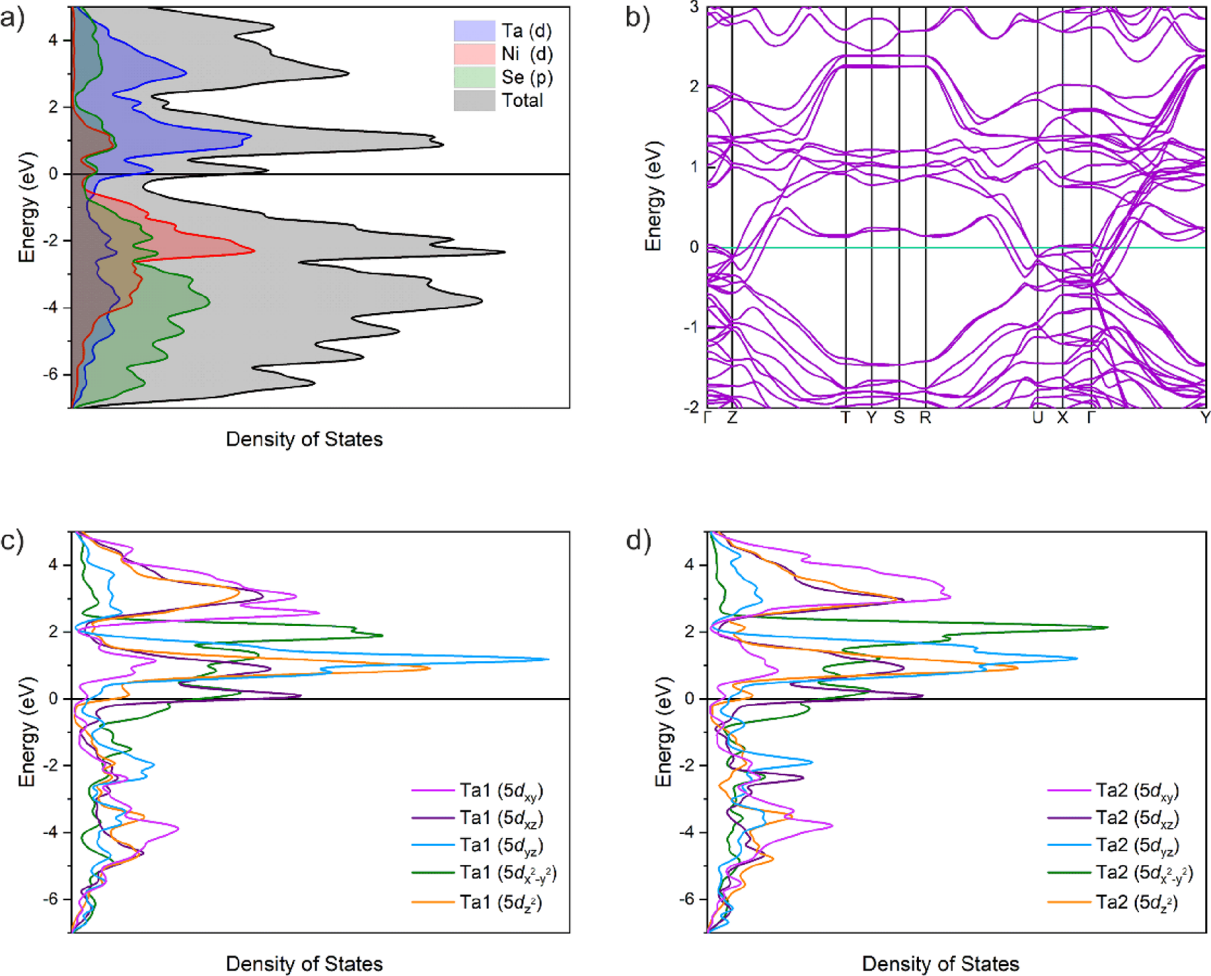
(a) Density of states and (b) band dispersion of LiTa_2_NiSe_5_ calculated using the PBEsol functional. Partial density of states calculated for (c) Ta1 site and (d) Ta2 site in LiTa_2_NiSe_5_.

The electronic density of states (DOS) and band dispersion of LiTa_2_NiSe_5_ were calculated using the lowest-energy Li site given by DFT calculations and are shown in Figure 8a,b. The band dispersion shows no band gap and suggests that LiTa_2_NiSe_5_ is metallic. The DOS shows that the Ta 5d bands dominate above the Fermi level and are partially filled upon intercalation, which is consistent with partial reduction of Ta(V). Partial densities of states for the Ta1 and Ta2 (Figure 8c,d) sites show no significant difference between the two sites in the positions of the various bands relative to the Fermi Level, suggesting that reduction of Ta is not specific to one site. The small differences in partial densities of states presumably arise from the small difference in local coordination between the two Ta sites discussed above. The band structure and DOS of Ta_2_NiSe_5_ in both orthorhombic and monoclinic symmetries were calculated using the dispersion-corrected functionals PBEsol + D3 and are given in Figure S6 confirming the narrow-gap behavior quite different from the intercalate. We should note that the differences observed in the band structures and DOS between the parent phase and LiTa_2_NiSe_5_ are not simply due to changing the Fermi level by adding electrons donated from the intercalated Li but also due to the structural change described above that occurs during intercalation.

### NMR Spectroscopy

**Figure 9:**
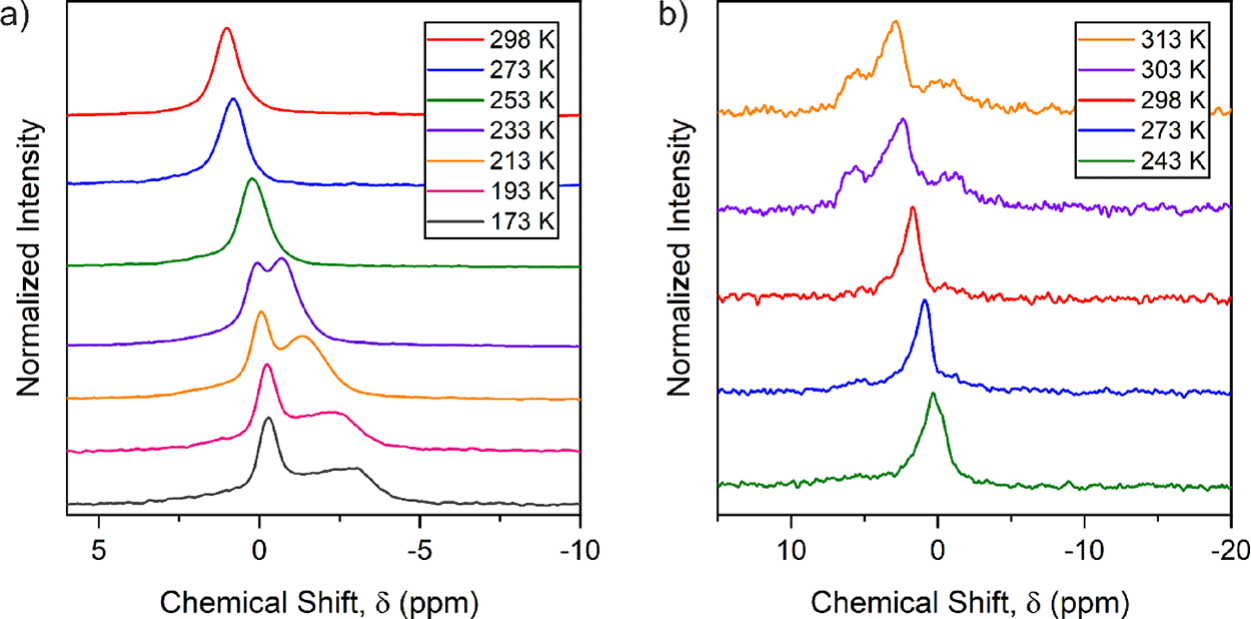
Variable-temperature MAS ^6^Li NMR spectra. (a) Low-temperature measurements between 173 and 298 K. (b) High-temperature measurements from 298 to 313 K. The two ranges are presented separately as they were performed as two measurements.

Variable-temperature MAS ^6^Li NMR measurements (Figure 9a) indicate a single Li environment at 298 K, observable on the NMR timescale, which agrees with our previous Li analysis. However, low-temperature measurements reveal the appearance of a second asymmetric peak between 253 and 233 K, which contains the majority of the Li and becomes extremely broad upon cooling. This could be due to a second Li environment, although none of the higher-energy Li sites located computationally refined to nonzero occupancies outside the uncertainty in the parameters in the PND structure refinements. The nature of the splitting is unusual: one peak shifts and broadens significantly relative to the other. This could indicate a temperature-dependent Knight shift usually observed in metallic compounds arising from the Pauli paramagnetism of conduction electrons^48^ and is proportional to the electronic density of states at the Fermi level.

Above room temperature (Figure 9b), two additional peaks appear, which could indicate the appearance of a conductive phase above 298 K, as indicated by spinning problems and changes in probe tuning, although no structural transition or decomposition is apparent in the variable-temperature PXRD data until much higher temperatures, as described above. Variable-temperature conductivity measurements are hampered by the air sensitivity of this compound, although the calculations and a crude resistivity measurement (see below) suggest metallic behavior for this intercalate.

Due to the lack of implementation for computing the NMR shifts for metallic systems using ab initio methods, we attempted to calculate the NMR shifts with the gauge-including projector augmented wave, an approach designed for nonmetallic crystalline systems.^49−51^ The calculations were unsuccessful due to the metallic nature of the compound. Detailed methodology can be found in the Supporting Information.

### Magnetometry

Ambient-temperature magnetization isotherms measured using SQUID magnetometry reveal that the parent and intercalate compounds are bulk diamagnets. At low fields, both isotherms reveal small positive magnetizations from 0 to 1 T. This is indicative of a small ferromagnetic impurity and has been attributed to the presence of elemental Ni in trace amounts (0.0205(8) and 0.0106(2)%, respectively) below the detection limit of X-ray and neutron powder diffraction. The susceptibility was determined as a function of temperature by performing measurements at two fields above the saturation field of the ferromagnetic impurity.

**Figure 10:**
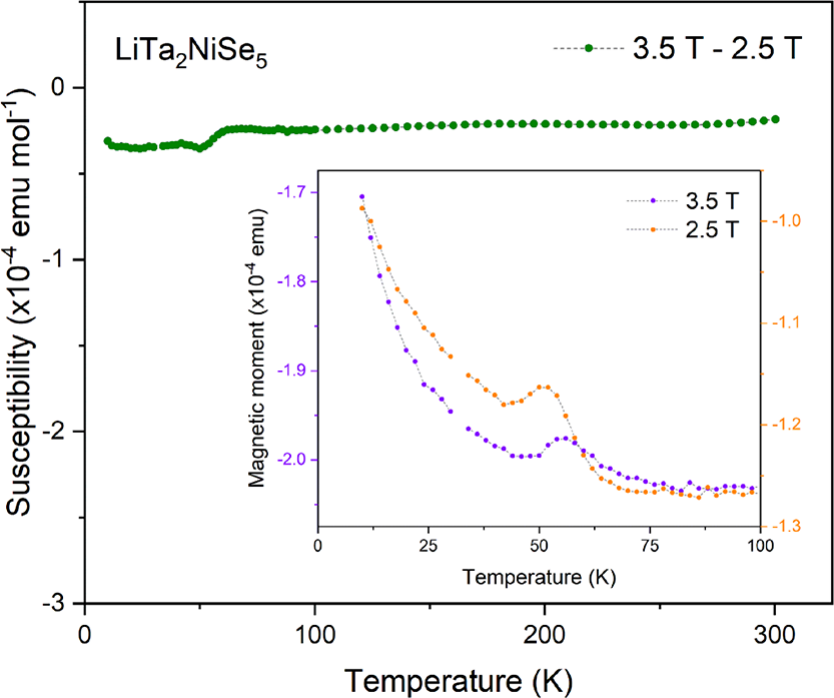
Molar susceptibility vs temperature curves collected at 2.5 and 3.5 T after being cooled in zero field. The green curve is the subtraction of the data points collected at 3.5 T from the data points collected at 2.5 T. The hump at approximately 50 K has been attributed to a trace amount of oxygen contamination.

Figure S7 shows a 30% reduction in the diamagnetic susceptibility, from −1.695(7) × 10^–4^ emu mol^–1^ in Ta_2_NiSe_5_ (DiSalvo *et al*. reported a susceptibility of −1.028 × 10^–4^ emu mol^–1^ for Ta_2_NiSe_5_ at 300 K^19^) to −1.254(4) × 10^–4^ emu mol^–1^ in LiTa_2_NiSe_5_, upon intercalation. Subtraction of the magnetization vs temperature curve measured at 2.5 T from that measured at 3.5 T (Figure 10) for LiTa_2_NiSe_5_ eliminates the effect of the minuscule ferromagnetic impurity and gives the intrinsic susceptibility, which is temperature-independent, consistent with opposing temperature-independent diamagnetic and paramagnetic contributions. The core diamagnetic contributions calculated from standard tables^52^ are −2.8 × 10^–4^ and −2.81 × 10^–4^ emu mol^–1^ for Ta_2_NiSe_5_ and LiTa_2_NiSe_5_, respectively. However, the experimental susceptibilities are approximately half of these values. This suggests that there is a small temperature-independent paramagnetism in the parent Ta_2_NiSe_5_ and the intercalate is a Pauli paramagnet with a temperature-independent susceptibility enhanced by the injection of electrons from the intercalated Li to increase the density of states at the Fermi level. This is consistent with the clear conclusion from the calculations that the intercalated phase LiTa_2_NiSe_5_ is metallic. Conductivity measurements on Ta_2_NiSe_5_ have been reported previously by DiSalvo *et al.*(19) Similar measurements of LiTa_2_NiSe_5_ were hampered by the air sensitivity of the sample. A room temperature measurement was made under nonideal conditions inside a glovebox by sandwiching a 5 mm-diameter cold-pressed pellet of LiTa_2_NiSe_5_ between two steel contacts of the same diameter, which were connected to a multimeter, which gave an estimate of the resitivity, ρ, of 2.1(1) Ω cm, consistent with metallic behavior.

No superconductivity was observed in LiTa_2_NiSe_5_ down to 2 K (the lower limit of our instrument) at ambient pressure in a separate magnetometry experiment using an applied field of 50 Oe. While the arrangement of the layers in the intercalated phase is similar to that in the high-pressure form of Ta_2_NiSe_5_, which exhibits superconductivity below 1.2 K at a pressure of approximately 8 GPa,^28^ the intercalation provides additional electrons to raise the Fermi level. Whether superconductivity is present in LiTa_2_NiSe_5_ below 2 K, at elevated pressure, or at different levels of intercalation (if attainable) is not predictable and may be a suitable subject for further experiments.

## Conclusions

We have successfully synthesized and characterized metallic-phase LiTa_2_NiSe_5_ synthesized by the intercalation of Li into the narrow-band-gap excitonic insulator candidate Ta_2_NiSe_5_ using *n*-butyllithium, showing that a crystalline intercalate is obtainable in this system.^30^ Displacement of the Ta_2_NiSe_5_ layers relative to one another occurs during intercalation, without a significant loss of crystallinity, and is accompanied by a significant increase in the unit cell volume of 5.44(1)%. The resulting phase crystallizes in the orthorhombic space group *Pmnb* at room temperature, with no evidence of structural distortions detected between 100 and 500 K, in contrast to the case in the host phase Ta_2_NiSe_5_. Li has been found to fully occupy triangular prismatic sites with an average Li–Se bond length of 2.724(2) Å. SQUID magnetometry shows that LiTa_2_NiSe_5_ is also diamagnetic but shows a significant reduction in magnetic susceptibility due to the enhancement of temperature-independent paramagnetism by injection of electrons from Li to increase the density of states at the Fermi level. No sign of superconductivity was found down to 2 K at ambient pressure. ^6^Li NMR studies reveal unusual behavior at low temperatures and a possible electronic transition just above room temperature. Further investigation is needed to rationalize these observations.

## Author Information

P.A.H. synthesized the samples and analyzed the experimental data with help from S.J.Ca in structure determination. R.I.S collected the neutron diffraction data. J.C. performed the calculations under the direction of B.Z. and D.O.S. P.H. performed the chemical analysis. N.H.R. performed the NMR experiments. P.A.H. wrote the paper with input from the other authors. S.J.Cl. provided materials and initial concepts and oversaw the analysis.

## Supporting Information Available

Further diffractograms, refinement parameters, and magnetometry and conductivity data (PDF)
