# Supplementary material for: Lithium Intercalation into the Excitonic Insulator Candidate Ta2NiSe5
Source: Inorg Chem. 2023 Jul 19;62(30):12027–37. doi: 10.1021/acs.inorgchem.3c01510 (PMC10394660; doi:10.1021/acs.inorgchem.3c01510)
Supplement: Supplementary file 1 — ic3c01510_si_001.pdf [file ic3c01510_si_001.pdf]

Lithium Intercalation into the Excitonic Insulator Candidate Ta_2NiSe_5

P. A. Hyde,[a] J. Cen,[b,c] S. J. Cassidy,[a] N. H. Rees,[a] P. Holdship,[d] R. I. Smith,[e] B. Zhu,[b,c] D. O. Scanlon,[b,c] S. J. Clarke[a]*

[a]*Department of Chemistry, University of Oxford, Inorganic Chemistry Laboratory, South Parks Road, Oxford OX1 3QR, UK*

[b]*Department of Chemistry, University College London, 20 Gordon Street, London, WC1H 0AJ, UK*

[c]*Thomas Young Centre, University College London, Gower Street, London, WC1E 6BT, UK*

[d]*Department of Earth Sciences, University of Oxford, South Parks Road, Oxford, OX1 3AN, UK*

[e]*ISIS Facility, Rutherford Appleton Laboratory, Harwell Campus, Didcot, Oxon, OX11 0QX, UK*

Supporting Information

*email address: simon.clarke@chem.ox.ac.uk

Table S1. Experimental and refined parameters of $LiTa_2NiSe_5$ from the PXRD pattern collected on the I11 beamline and the PND pattern collected on GEM.

	**$LiTa_2NiSe_5$ RMM = 822.33 g mol^{-1}, Z = 4**	
Diffractometer	GEM (ISIS)	I11 (PSD)
Wavelength (Å)	0.5-4.2 (White beam TOF)	0.82445(2)
Radiation	Neutron	X-ray
d-space range	0.11-25	0.57-22.7
Temperature (K)	300	300
Crystal system	Orthorhombic	
Space group	*Pmnb* (62)	
a (Å)	3.49523(6)	3.50247(3)
b (Å)	13.3681(3)	13.4053(4)
c (Å)	15.7015(4)	15.7396(2)
V (Å^3)	733.644(28)	739.002(27)
χ^2	0.0110	44.514
R_p	3.463	1.138
R_{wp}	4.584	1.759

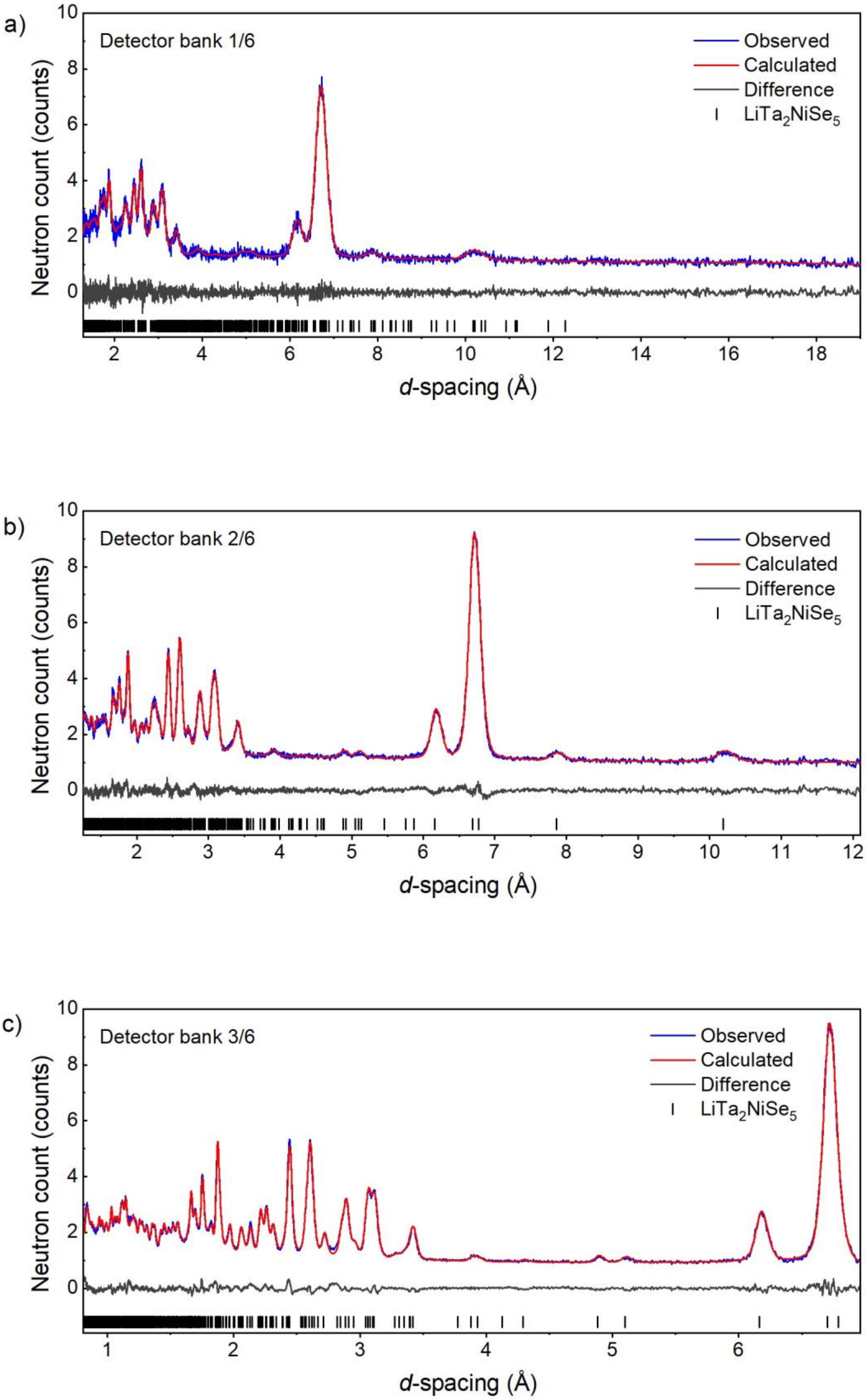
a)
Detector bank 1/6
Observed
Calculated
Difference
$LiTa_2NiSe_5$
Neutron count (counts)
d-spacing (Å)
b)
Detector bank 2/6
Observed
Calculated
Difference
$LiTa_2NiSe_5$
Neutron count (counts)
d-spacing (Å)
c)
Detector bank 3/6
Observed
Calculated
Difference
$LiTa_2NiSe_5$
Neutron count (counts)
d-spacing (Å)

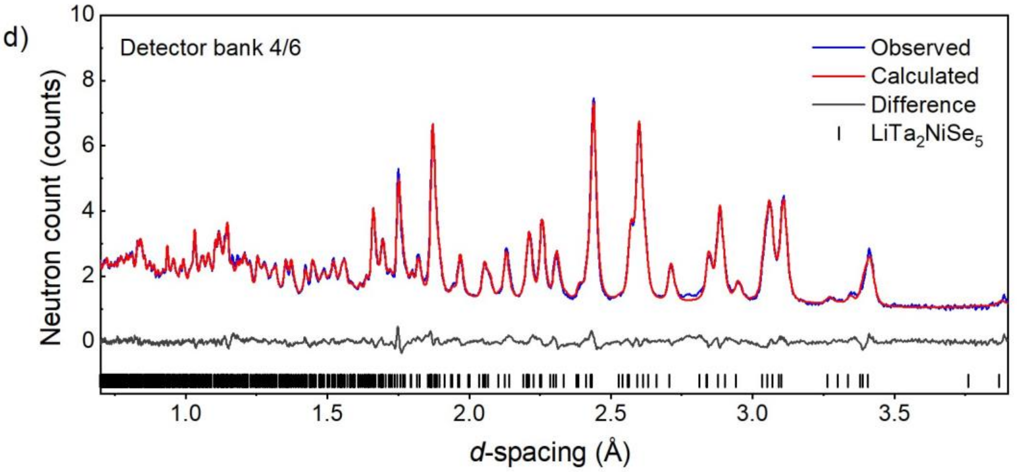

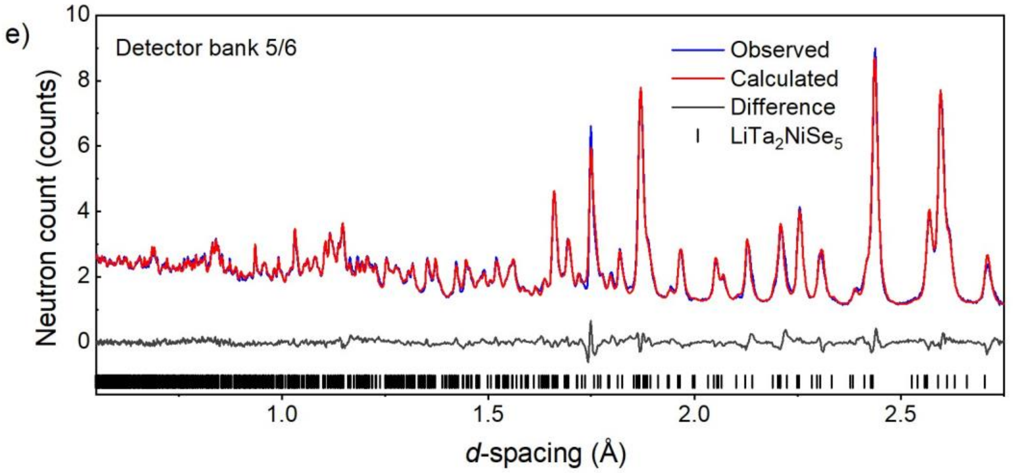

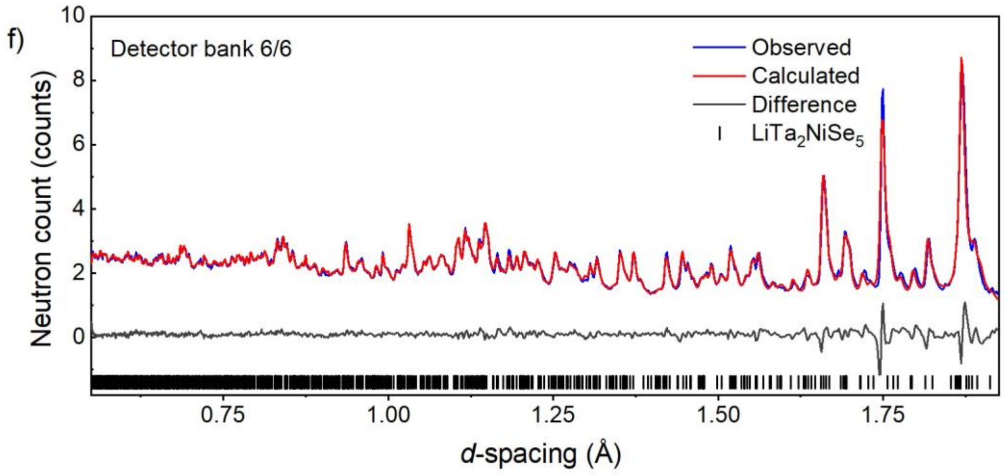

Figure S1. Rietveld fits to PND data collected in all six banks of GEM at ISIS, UK.

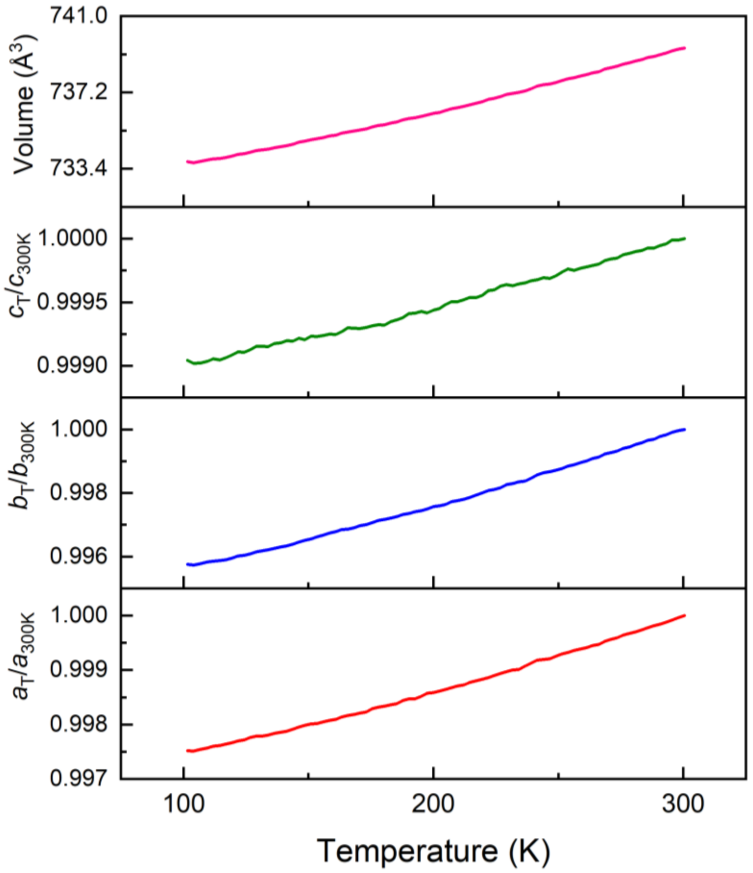

Figure S2. Lattice parameter variation from temperature resolved PXRD measurements from 100 K to 300 K, using the PSD detector on beamline I11 at Diamond Light Source, UK.

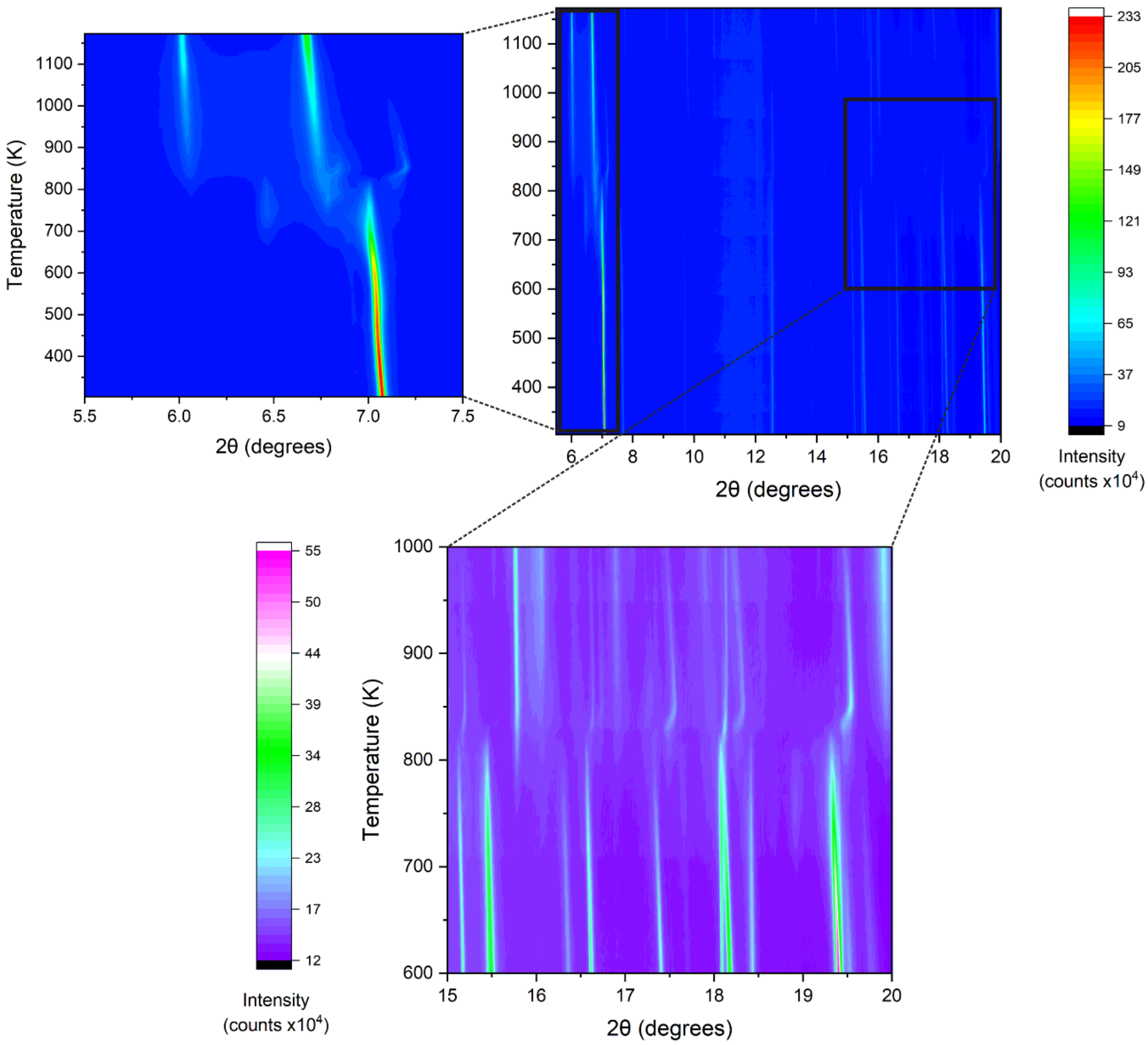

Figure S3. Temperature resolved PXRD data collected on the I11 beamline between 303 – 1173 K over 211 temperatures.

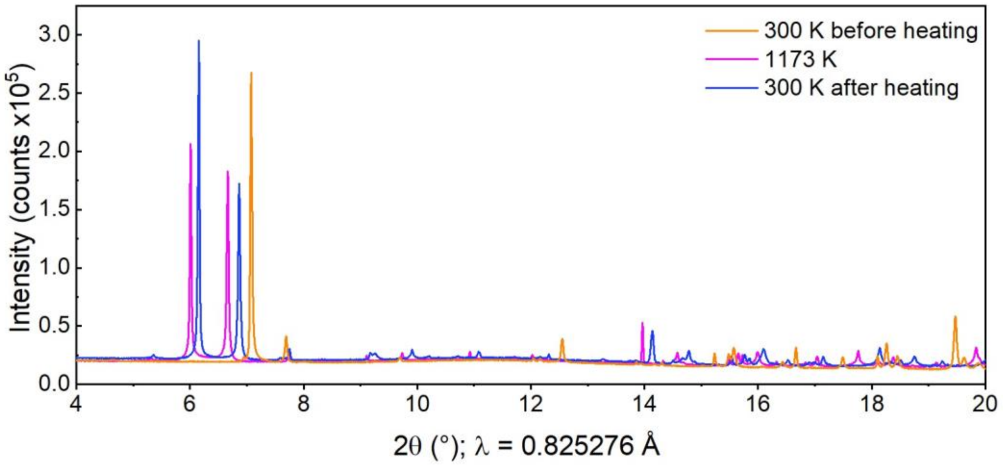

Figure S4. PXRD patterns measured at 300 K, 1173 K and 300 K after heating using the PSD detector on beamline I11 at Diamond Light Source, UK. The patterns collected at 1173 K and 300 K post heating may not be fit by known phases and may represent a high-temperature phase of $LiTa_2NiSe_5$ that is preserved on cooling or an unidentified mixture of decomposition products.

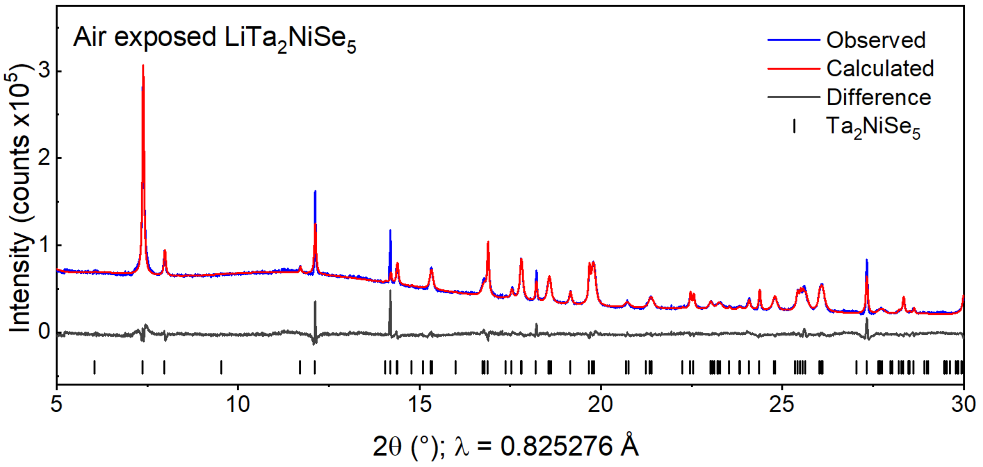

Figure S5. Rietveld fit to air exposed $LiTa_2NiSe_5$ PXRD data collected using the PSD detector on beamline I11 at Diamond Light Source, UK. R_{wp} = 4.69 %

Table S2. Parameters of relaxed interstitial sites output by plane wave density functional theory calculations. Each site can be attributed to one of five 'clusters' of sites which occupy the same region in the unit cell. The site marked with an asterisk (*) was the only one not located on a mirror plane in the *Pmnb* model and in the relaxation of the initial model, the symmetry was reduced to $P2_1/c$ to allow it to fully occupy a site of 4-fold multiplicity. ΔE is the total energy difference relative to the lowest energy cluster model per unit cell used in the calculation. Li site energy is ΔE normalised by the number of Li atoms in the unit cell.

Defect Name	ΔE (eV)	Site	Li Site Energy (eV per Li)	Site co-ordinates		
				x	y	z
**Cluster 1**						
Int_Li_14	0	4*c*	0	0.25	0.50114	0.33392
Int_Li_2	0	4*c*	0	0.25	0.48885	0.33518
Int_Li_12	0	4*c*	0	0.25	0.49714	0.33272
Int_Li_16	0	4*c*	0	0.25	0.50199	0.33267
Int_Li_18	0	4*c*	0	0.25	0.49362	0.33384
Int_Li_17	0	4*c*	0	0.25	0.49384	0.33157
Int_Li_15	0	4*c*	0	0.25	0.50124	0.33008
Int_Li_13	0.01	4*c*	0	0.25	0.49654	0.32908
**Cluster 2**						
Int_Li_8	0.08	4*c*	0.02	0.25	0.46758	0.07313
Int_Li_9	0.08	4*c*	0.02	0.25	0.46977	0.07400
Int_Li_7*	0.08	4*e*	0.02	0.25735	0.46595	0.07039
Int_Li_6	0.08	4*c*	0.02	0.25	0.46429	0.06736
Int_Li_5	0.08	4*c*	0.02	0.25	0.46220	0.06621
**Cluster 3**						
Int_Li_10	0.15	4*c*	0.04	0.25	0.50076	0.92858
Int_Li_4	0.15	4*c*	0.04	0.25	0.50964	0.92808
Int_Li_3	0.15	4*c*	0.04	0.25	0.50484	0.92916
Int_Li_19	0.15	4*c*	0.04	0.25	0.49277	0.92998
Int_Li_11	0.15	4*c*	0.04	0.25	0.50017	0.92949
**Cluster 4**						
Int_Li_21	0.46	4*c*	0.11	0.25	0.57913	0.74604
Int_Li_20	0.46	4*c*	0.11	0.25	0.58248	0.74324
Int_Li_22	0.46	4*c*	0.11	0.25	0.57631	0.74609
**Cluster 5**						
Int_Li_1	0.68	4*c*	0.17	0.25	0.54079	0.51680

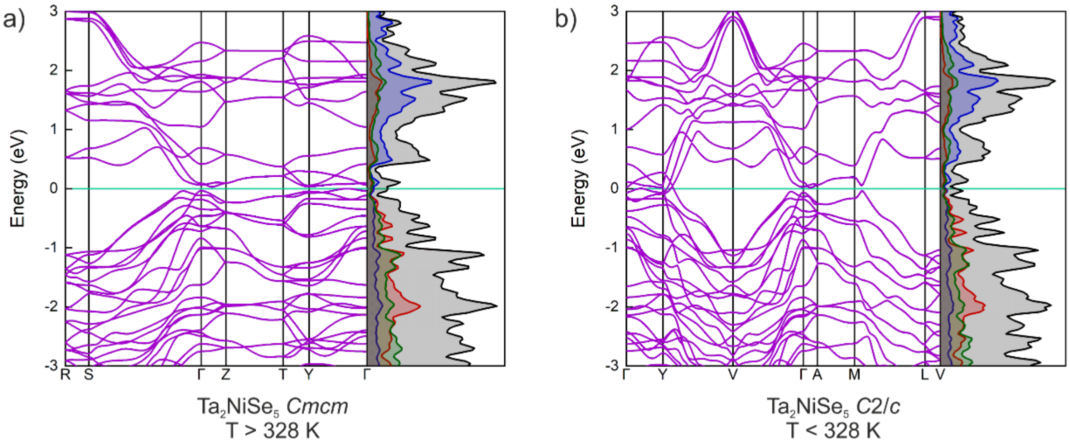

Figure S6. The band dispersion and density of states for Ta_2NiSe_5 in (a) orthorhombic (*Cmcm*) symmetry and (b) in monoclinic (*C*2/*c*) symmetry were computed using dispersion corrected PBEsol + D3 functionals.

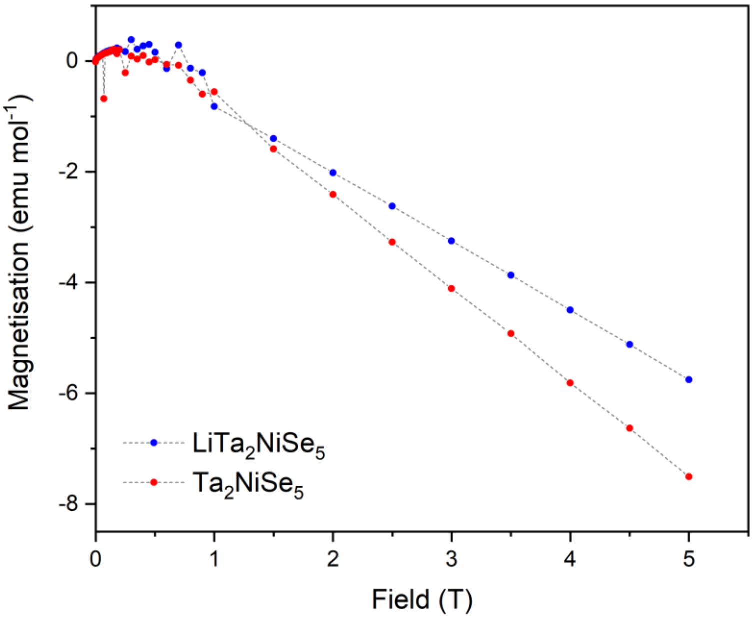

Figure S7. Magnetisation vs field for Ta_2NiSe_5 (red) and $LiTa_2NiSe_5$ (blue) at 300 K. Magnetic impurities attributed to elemental Ni can be seem at low field but saturate above 1.5 T. Linear fits of the high field region show both phases are diamagnetic. Magnetic susceptibility was calculated by taking the gradient in this region resulting in values of -1.695(7) $\times 10^{-4}$ and -1.254(4) $\times 10^{-4}$ emu mol^{-1} for Ta_2NiSe_5 and its intercalate respectively. Ni impurities were found to be 0.0205(8) % and 0.0106(2) % respectively.

NMR Calculation Methodology

Calculation of NMR parameters was attempted using the gauge-including projector augmented-wave (GIPAW) approach in CASTEP[1–3] (version 19.11). Lithiated structures were subject to geometry optimisation with the default LBFGS method prior to NMR calculations. Ultrasoft pseudopotentials [4,5] were generated on the fly as defined in the built-in C19 library and GGA-PBE [6] exchange-correlation functional was used as it is the most widely used functional in GIPAW calculations. [7] A plane wave cutoff energy of 700 eV was used with MP k-point grids with a maximum spacing of 0.04 2π $Å^{-1}$. The calculations generated absolute magnetic shielding tensors and the MagresPython[8,9] library was used for computing the chemical shift values with LiCl referenced at -1 ppm.

(1) Clark, S. J.; Segall, M. D.; Pickard, C. J.; Hasnip, P. J.; Probert, M. I. J.; Refson, K.; Payne, M. C. First Principles Methods Using CASTEP. *Z. Krist. Cryst. Mater* **2005**, *220*, 567–570.

(2) Pickard, C. J.; Mauri, F. All-Electron Magnetic Response with Pseudopotentials: NMR Chemical Shifts. *Phys. Rev.* **2001**, *63*, 245101. https://doi.org/10.1103/PhysRevB.63.245101.

(3) Yates, J. R.; Pickard, C. J.; Mauri, F. Calculation of NMR Chemical Shifts for Extended Systems Using Ultrasoft Pseudopotentials. *Phys. Rev. B* **2007**, *76*, 024401. https://doi.org/10.1103/PhysRevB.76.024401.

(4) Vanderbilt, D. Rapid Communications Soft Self-Consistent Pseudopotentials in a Generalized Eigenvalue Formalism. *Phys. Rev. B* **1990**, *41*, 7892–7895.

(5) Louie, S. G.; Froyen, S.; Cohen, M. L. Nonlinear Ionic Pseudopotentials in Spin-Density-Functional Calculations. *Phys. Rev. B* **1982**, *26*, 1738. https://doi.org/10.1103/PhysRevB.26.1738.

(6) Perdew, J. P.; Burke, K.; Ernzerhof, M. Generalized Gradient Approximation Made Simple. *Phys. Rev. Lett.* **1996**, *77*, 3865.

(7) Bonhomme, C.; Gervais, C.; Babonneau, F.; Coelho, C.; Frédé, F.; Pourpoint, F.; Azaïs, T.; Ashbrook, S. E.; Griffin, J. M.; Yates, J. R.; Mauri, F.; Pickard, C. J. First-Principles Calculation of NMR Parameters Using the Gauge Including Projector Augmented Wave Method: A Chemist's Point of View. *Chem. Rev.* **2012**, *112*, 5733–5779. https://doi.org/10.1021/cr300108a.

(8) Green, T. *magres-format*. https://github.com/tfgg/magres-format.

(9) Sturniolo, S.; Green, T. F. G.; Hanson, R. M.; Zilka, M.; Refson, K.; Hodgkinson, P.; Brown, S. P.; Yates, J. R. Visualization and Processing of Computed Solid-State NMR Parameters: MagresView and MagresPython. *Solid State Nucl. Magn. Reson.* **2016**, *78*, 64–70. https://doi.org/10.1016/J.SSNMR.2016.05.004.

Note for Table S1.

In the Rietveld refinement the function s_y is minimised

$$S_y = \sum_i w_i (y_i - y_{ci})^2$$

where y_i is the observed, and y_{ci} the calculated intensity at point *i* and w_i is the weighting factor, defined by $\frac{1}{y_i}$

The weighted profile *R* factor, R_{wp} is

$$R_{wp} = \sqrt{\frac{\sum_i w_i (y_i - y_{ci})^2}{\sum_i w_i y_i^2}}$$

The profile R factor, R_p is

$$R_p = \sqrt{\frac{\sum_i |y_i - y_{ci}|}{\sum_i y_i}}$$

The statistically expected *R* value, R_{exp}, in which all deviations of the calculated pattern from the observed are due to statistical variations. R_{exp} is defined by:

$$R_{exp} = \sqrt{\frac{N_{obs} - N_{var}}{\sum_i w_i y_i^2}}$$

where N_{obs} and N_{var} are the number of observables and number of variables respectively.

A goodness of fit parameter, χ^2, is defined from the square of the ratio of R_{wp} and R_{exp}.
